# Integrating Network Pharmacology and Experimental Validation to Explore the Effect and Mechanism of *Inonotus obliquus* Polysaccharide in the Treatment of Rheumatoid Arthritis

**DOI:** 10.3390/ph18071017

**Published:** 2025-07-08

**Authors:** Yuan Fu, Tianyi Jiang, Xizhu Fang, Yifang Chen, Jiawei Li, Shengnan Huang, Fangfang Li, Dan Jin

**Affiliations:** 1 Immunology Biology Key Laboratory, Yanbian University, Yanji 133002, China; yfu1990@126.com (Y.F.); jianglate2025@163.com (T.J.); 0000007941@ybu.edu.cn (X.F.); chenyifang@ioz.ac.cn (Y.C.); lijiawei@imun.edu.cn (J.L.); 15053791276@163.com (S.H.); 2 Key Laboratory of Natural Medicines of the Changbai Mountain, Ministry of Education, Yanbian University, Yanji 133002, China; 3 Department of Immunology and Pathogenic Biology, College of Medicine, Yanbian University, Yanji 133002, China

**Keywords:** *Inonotus obliquus* polysaccharide, rheumatoid arthritis, network pharmacology, NF-κB pathway, NLRP3 inflammasome

## Abstract

**Background/Objectives:** Rheumatoid arthritis (RA) is a chronic, systemic, and progressive autoimmune–inflammatory disease primarily affecting small joints. *Inonotus obliquus* polysaccharide (IOP) is the main component of the parasitic fungus obliquus, which has anti-tumor, anti-inflammatory, and antioxidant effects. However, whether IOP has a therapeutic effect on RA is still unclear. Thus, this study aimed to reveal the effect of IOP on MH7A cells and collagen-induced arthritis (CIA) rats and to investigate the molecular mechanism of IOP in RA. **Methods:** In this study, network pharmacology was used to identify the key signaling pathways in IOP treatment of RA. The effect of IOP was verified in rats with CIA. We performed CCK-8, EdU, colony formation assay, cell apoptosis, cell migration and invasion, Western blot analysis, and immunofluorescence to elucidate the effect of IOP on the proliferation, apoptosis, migration and invasion of MH7A cells and revealed its modulation of the NF-κB and NLRP3 inflammasome signaling pathways. **Results:** IOP treatment of CIA rats significantly alleviated joint swelling, synovial tissue proliferation and erosion, and reduced the expression of inflammatory factors TNF-α, IL-6, IL-1β and IL-18. In vitro, IOP significantly inhibited the proliferation, migration, and invasion abilities of TNF-α-stimulated MH7A cells and promoted their apoptosis. Mechanistically, IOP inhibited the NF-κB and NLRP3 inflammasome activation. **Conclusions:** This study revealed that IOP exerts anti-RA effects by downregulating the NF-κB and NLRP3 inflammasome signaling pathways, promoting cell apoptosis, and inhibiting the expression of inflammatory cytokines, representing a promising therapeutic option for RA.

## 1. Introduction

Rheumatoid arthritis (RA) is a chronic, systemic, autoimmune inflammatory disease that mainly affects the joints and periarticular soft tissues [1]. It affects 0.3% to 1% of the worldwide population, and its typical symptoms include joint discomfort, edema, morning stiffness, and restricted movement [2]. It has been demonstrated that cytokines, including tumor necrosis factor-α (TNF-α), interleukin (IL)-1β, IL-18, IL-6, and other inflammatory mediators, play crucial roles in the pathogenesis of RA [3,4,5]. Although treatments such as methotrexate (MTX), nonsteroidal anti-inflammatory drugs, disease-modifying antirheumatic drugs (DMARDs), and glucocorticoids (GCs) are clinically used for RA, their therapeutic effects are far from satisfactory and are often associated with severe side effects [6]. Therefore, it is essential to develop a therapeutic agent that is well-tolerated, has minimal side effects, and maintains consistent efficacy.

In the synovium of RA patients, nuclear factor kappa B (NF-κB) promotes the proliferation of synovial fibroblasts and stimulates osteoclast formation, which in turn increases the expression of inflammatory factors such as IL-1α, IL-1β, IL-6, IL-17, and TNF-α, leading to bone destruction [7,8,9,10]. Furthermore, NF-κB can activate the NOD-like receptor family pyrin domain containing 3 (NLRP3) inflammasome via nuclear transcription. Activation of NLRP3 regulates various inflammatory factors under inflammatory and stress conditions, promoting fibroblast proliferation and activation [11]. Accordingly, inhibition of NLRP3 inflammasome activity can effectively mitigate the inflammatory process and alleviate RA symptoms [12].

*Inonotus obliquus* is an edible fungus containing various chemical components, including polysaccharides, triterpenoids, and polyphenols. Long-term clinical and animal experiments have shown that it possesses anticancer, anti-inflammatory, antiviral, antioxidant, hypoglycemic, and hypolipidemic activities without notable side effects [13,14]. Among these, *Inonotus obliquus* polysaccharide (IOP) has been demonstrated to exhibit anti-tumor [15,16], anti-inflammatory [17], antioxidant [18], and antiparasitic [19] properties. Mishra et al. found that IOP reduced the inflammatory mediators TNF-α, inducible nitric oxide synthase (iNOS), and IL-1β, thereby preventing acute enteritis [17]. Similarly, Park et al. reported that IOP exerted a strong analgesic effect in the rat model of acute foot edema, and could reduce the expression of nitric oxide, prostaglandin, TNF-α, and iNOS through the NF-κB pathway to alleviate inflammation [20]. In addition, our previous study found that IOP mitigated the inflammatory symptoms of dextran sulfate sodium (DSS)-induced colitis in mice by regulating the JAK-STAT signaling pathway and suppressing the levels of pro-inflammatory cytokines in activated T cells to balance T helper cell 1 (Th1)/Th2 and Th17/Treg functions [21]. While IOP has shown substantial anti-inflammatory effects in various models, its therapeutic potential and underlying mechanisms in RA have not been fully explored.

Based on prior evidence that IOP exerts anti-inflammatory effects in models of colitis and acute inflammation [17,20,21], we hypothesize that IOP can ameliorate RA by modulating key inflammatory signaling pathways. The objective of this study is to integrate network pharmacology with experimental validation to elucidate the molecular targets and mechanisms underlying IOP’s therapeutic potential in RA. We first employed network pharmacology to predict IOP’s molecular targets in RA and used protein–protein interaction (PPI) and pathway enrichment analyses to identify critical pathways. These predictions were then validated in vitro using TNF-α–stimulated MH7A synoviocytes and in vivo in a collagen-induced arthritis (CIA) rat model. Taken together, these findings provide a comprehensive framework for understanding IOP’s mechanism of action in RA and support its further development as a novel therapeutic candidate.

## 2. Results

### 2.1. Key Targets and Pathways of IOP in RA Identified by Network Pharmacology

**Figure 1:**
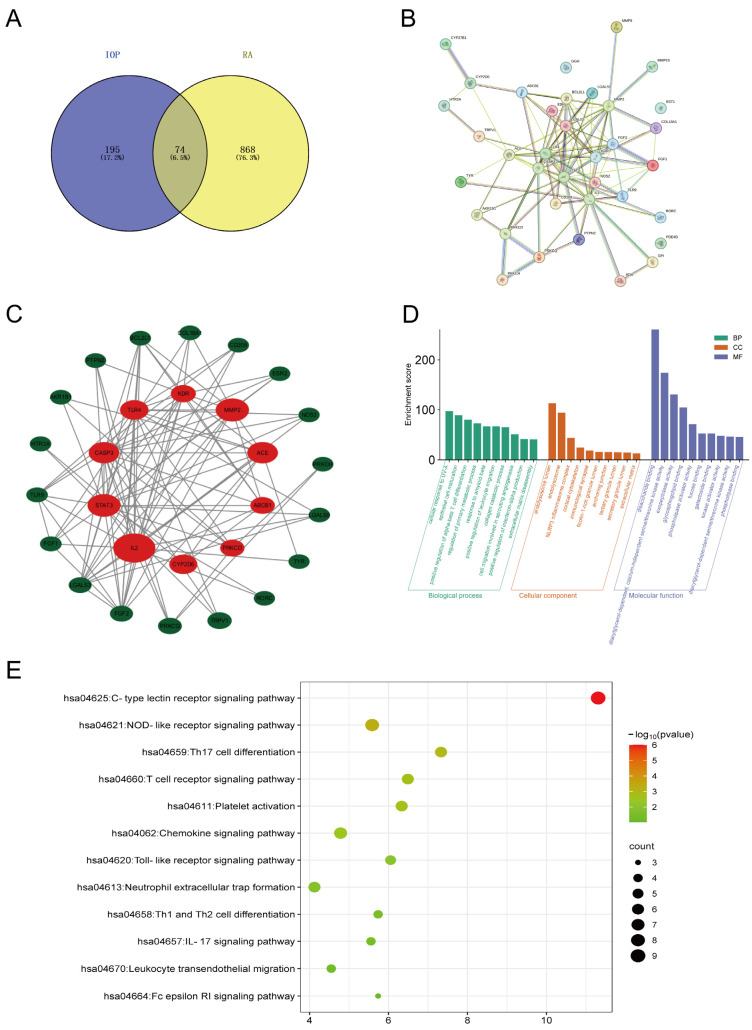
Network pharmacology analysis of IOP and RA. (**A**) Venn diagram showing 74 overlapping target proteins between IOP targets and related genes of RA. (**B**) PPI network of 36 cross-targets. (**C)** Key common targets related to both IOP and RA. (**D**) GO enrichment analysis, including BP, CC, and MF. (**E**) Bubble diagram of KEGG pathway enrichment.

To identify potential targets of IOP for RA, we uploaded the main molecular structure of IOP (Supplementary Figure S1) to PharmMapper and Swiss Target Prediction databases and identified 269 potential targets. In addition, a total of 942 RA-related genes were obtained from Online Mendelian Inheritance in Man (OMIM), GeneCards, and Therapeutic Target databases. IOP targets and RA-related genes were then uploaded to VENNY 2.1 to generate Venn diagrams (Figure 1A), identifying 74 intersecting genes as potential candidates for IOP targeting RA. To further explore the relationship between these overlapping genes, they were uploaded to the STRING database to construct the PPI network, consisting of 36 nodes and 110 edges (Figure 1B). We then uploaded the PPI network to Cytoscape software (v3.8.2) for visualization, and the CytoHubba plugin was used to identify the top 10 key genes by the degree method: *IL-2*, *STAT3*, *CASP3*, *TLR4*, *KDR*, *MMP2*, *ACE*, *ABCB1*, *PRKCD*, *CYP2D6* (Figure 1C and Table 1). Next, we performed GO enrichment analysis on the 74 intersecting genes using the DAVID database. The analysis revealed significant enrichment in 301 biological processes (BPs), 36 cellular components (CCs), and 66 molecular functions (MFs) of 74 anti-RA crossover genes of IOP. The top 10 enriched biological processes, cellular components, and molecular functions of the 74 intersecting genes are shown in Figure 1D. In addition, a total of 104 Kyoto Encyclopedia of Genes and Genomes (KEGG) pathways were selected by KEGG pathway analysis using the KOBAS database. The C-type lectin receptor signaling pathway, the NOD-like receptor signaling pathway, and Th17 cell differentiation were found to be the three most important pathways (Figure 1E). Several studies have shown that NLRP3 is highly correlated with the pathogenesis of RA. Experimental investigation showed that the expression of NLRP3 was positively correlated with the severity of arthritis in the synovium of CIA mice [22]. Similarly, mice with antigen-induced arthritis showed severe joint inflammation and increased expression of IL-1β and NLRP3 inflammasome in the synovial membrane [23]. These findings suggest that IOP may exert its therapeutic effects on RA by regulating the NLRP3 inflammasome and related inflammatory pathways.

### 2.2. IOP Alleviates the Severity of Arthritis in Rats with CIA

**Figure 2:**
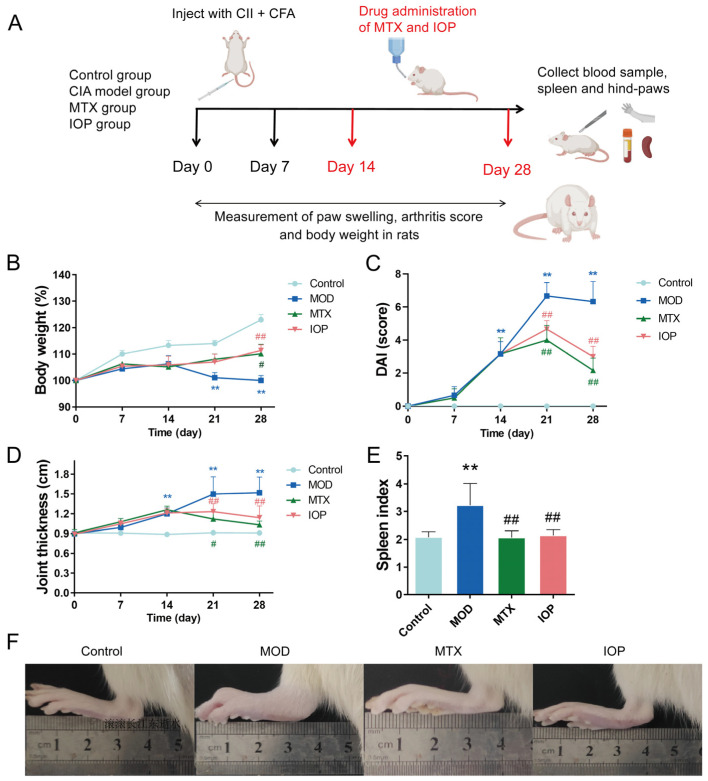

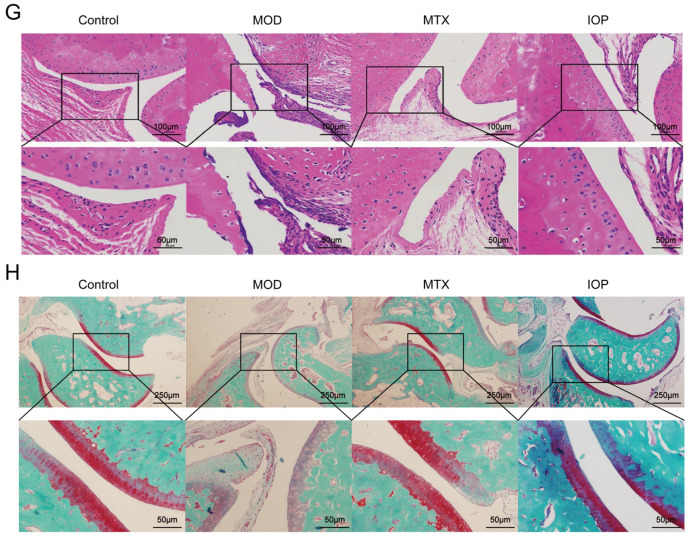
IOP restrains inflammatory responses and ameliorates symptoms of arthritis in CIA rats. The CIA rats were divided into control group, MOD group, MTX group, and IOP group. (**A**) A schematic of the experimental schedule for the establishment of the CIA model and drug treatment (MTX and IOP). (**B**–**E**) The changes in body weight, DAI score, paw swelling, foot, and spleen index of rats in each group during the animal experiment (n = 7). (**F**) Representative photographs of paws from each group. (**G**,**H**) H&E and safranin O-fast green staining of the ankle joint tissue of rats in each group, and the scores for inflammatory severity are also shown. Data are represented as mean ± SD. ** *p* < 0.01 vs. control group; ^#^ *p* < 0.05, ^##^ *p* < 0.01 vs. MOD group.

To evaluate the therapeutic effects of IOP on RA, we conducted in vivo experiments using the CIA rat model. Throughout the experiment (Figure 2A), control rats exhibited a continuous increase in body weight. In contrast, the MOD group showed significant weight loss commencing from day 21. However, this reduction was markedly reversed following treatment with MTX and IOP, indicating the therapeutic potential of IOP (Figure 2B). As shown in Figure 2C,D, during the whole experimental period, the DAI score and joint thickness of the MOD group increased notably from day 14 compared with the control group. Remarkably, treatment with MTX and IOP effectively reduced both the DAI scores and joint thickness from day 21, thereby alleviating the symptoms of arthritis. Furthermore, the spleen index was significantly elevated in the MOD group compared to the control group, but it was notably improved following treatment with MTX and IOP (Figure 2E). Macroscopic observations of the paws of CIA rats, recorded prior to pathological analysis, indicated symptom alleviation in groups treated with MTX and IOP (Figure 2F). This improvement was further demonstrated by histological evaluations, including H&E staining and safranin O-fast green staining (Figure 2G,H). Specifically, the MOD group exhibited severe synovial tissue hyperplasia and inflammatory cell infiltration, along with epithelial cell degradation. In contrast, the MTX and IOP treatment groups showed significantly mitigated abnormalities, characterized by reduced synovial tissue hyperplasia, erosion, and inflammatory cell infiltration. Therefore, these results indicated that IOP could relieve RA symptoms and improve histopathological inflammation.

### 2.3. IOP Inhibits the Expression of Inflammatory Mediators in CIA Rats

**Figure 3:**
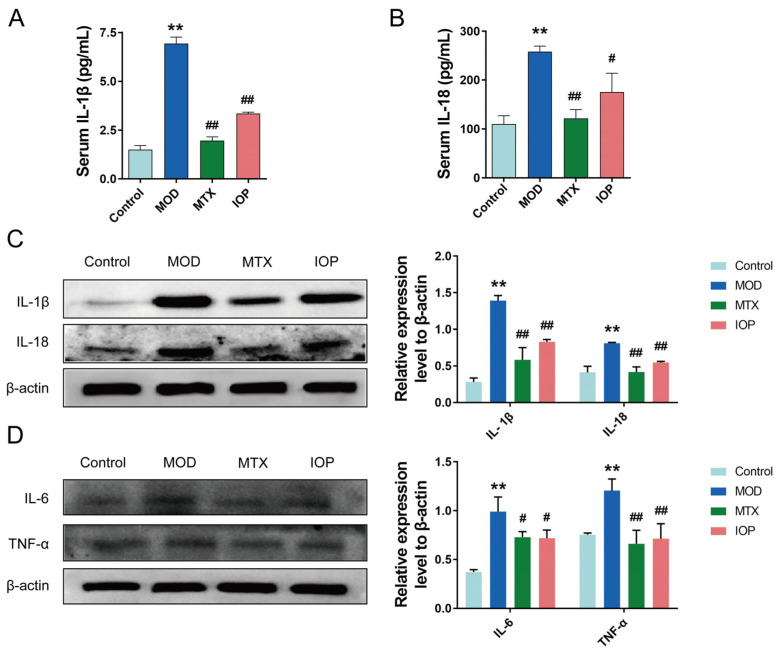
IOP significantly reduces the production of pro-inflammatory factors in serum and joints of CIA rats. (**A**,**B**) ELISA assay was conducted to assess the expression levels of IL-1β, IL-18 in the rats’ serum. (**C**,**D**) Western blot assay was performed to assess the protein expression levels of IL-1β, IL-18, IL-6, and TNF-α in the rats’ articular cartilage. Data are represented as mean ± SD. ** *p* < 0.01 vs. control group; ^#^ *p* < 0.05, ^##^ *p* < 0.01 vs. MOD group.

IL-1β, IL-18, IL-6, and TNF-α are vital endogenous factors involved in the pathogenesis of RA [24,25,26]. To further investigate the anti-inflammatory mechanisms of IOP, we examined the expressions of these critical pro-inflammatory cytokines in vivo. As shown in Figure 3A,B, compared with the control group, the expressions of IL-1β and IL-18 were significantly increased in the MOD group, and the serum cytokine levels were decreased after MTX and IOP treatment. We also found the protein levels of IL-1β, IL-18, IL-6, and TNF-α in the joints of rats in the MOD group (Figure 3C,D), but IOP treatment reduced their expression, similar to the effect of MTX.

### 2.4. IOP Inhibits NF-κB and NLRP3 Inflammasome Signaling Pathways in CIA Rats

**Figure 4:**
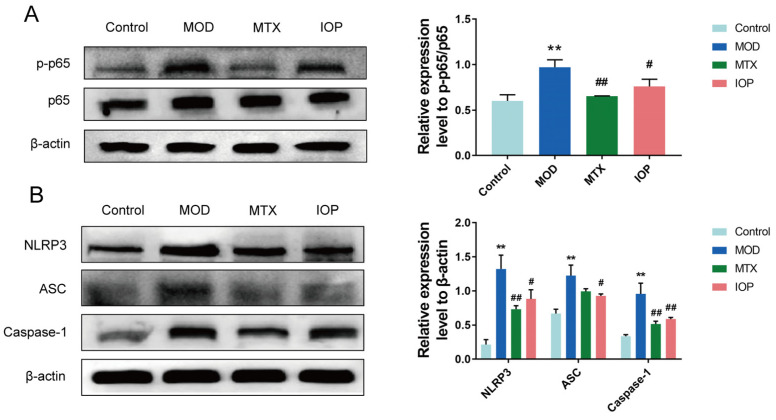
IOP inhibits the activation of NF-κB and NLRP3 pathways in the synovial membrane of CIA rats. (**A**,**B**) Western blot assay was conducted to assess the protein expression levels of NF-κB p-p65, NF-κB p65, NLRP3, ASC, and Caspase-1 in the articular cartilage of CIA rats. Data are represented as mean ± SD. ** *p* < 0.01 vs. control group; ^#^ *p* < 0.05, ^##^ *p* < 0.01 vs. MOD group.

To explore the underlying mechanism, we examined the NF-κB and NLRP3 inflammasome signaling pathways. Compared with the control group, the relative expression levels of NF-κB p-p65/p65, NLRP3, ASC, and Caspase-1 in the MOD group were significantly upregulated. However, MTX and IOP treatment inhibited the expression of these proteins (Figure 4A,B). Collectively, our data suggest that IOP treatment may attenuate the inflammatory response in arthritic rats by inhibiting the activation of the NF-κB and NLRP3 inflammasome signaling pathways.

### 2.5. IOP Affects Cell Viability and Proliferation of MH7A Cells

**Figure 5:**
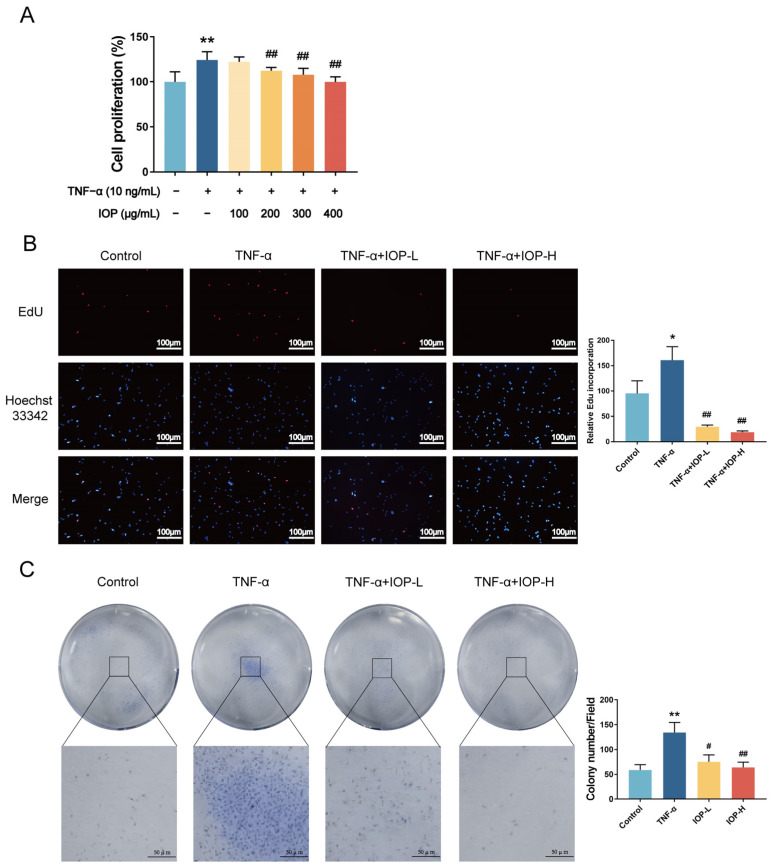
Effects of IOP on the viability and proliferation of MH7A cells. (**A**) The anti-proliferative effects of IOP (100, 200, 300, and 400 µg/mL) on MH7A cells stimulated with 10 ng/mL TNF-α was determined by CCK-8 analysis. (**B**) The DNA replication ability effects of IOP (200 and 400 µg/mL) in 10 ng/mL TNF-α-stimulated MH7A cells were detected by EdU assay (×100). (**C**) The effect of IOP (200 and 400 µg/mL) on the colony-forming ability of MH7A cells stimulated by TNF-α at 10 ng/mL was assessed using colony formation assay. Data are represented as mean ± SD. * *p* < 0.05, ** *p* < 0.01 vs. control group; ^#^ *p* < 0.05, ^##^ *p* < 0.01 vs. TNF-α group.

To further investigate the anti-RA mechanism of IOP, we performed in vitro experiments. The effect of IOP on the proliferation of MH7A cells was detected by CCK-8 assay. TNF-α (10 ng/mL) was used to stimulate cells, then IOP was added. The results showed that the TNF-α group had a significant proliferative effect on MH7A cells compared with the control group, and this effect was rescued by IOP (200, 300, and 400 μg/mL) in the 24 h treatment groups (Figure 5A). Based on these findings, the low-dose IOP group (200 μg/mL) and high-dose IOP group (400 μg/mL) were selected for further experiments. For further verification, we performed the EdU and colony-forming assays. The EdU assay showed that TNF-α significantly enhanced DNA synthesis in MH7A cells, but this was reversed by IOP treatment (Figure 5B). Similarly, the colony-forming assay demonstrated that TNF-α significantly enhanced the colony formation ability of MH7A cells, but this effect was inhibited by IOP. These results indicate that IOP had strong anti-proliferation activity.

### 2.6. IOP Inhibits Migration, Invasion and Induces Apoptosis of MH7A Cells

**Figure 6:**
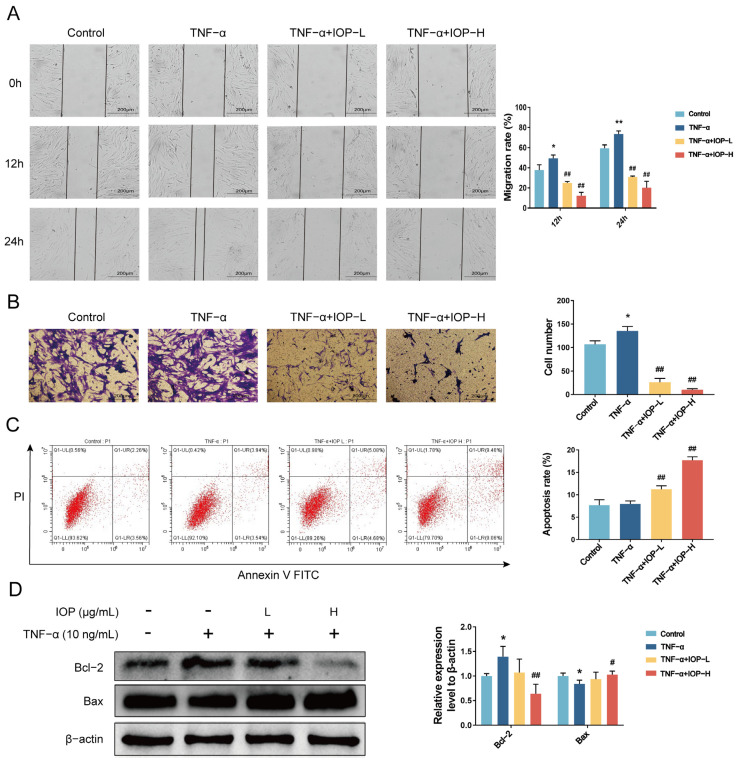
IOP inhibits migration and invasion and induces apoptosis of MH7A cells. MH7A cells were incubated with IOP (200 and 400 µg/mL) and pretreated with 10 ng/mL TNF-α. (**A**) The effect of IOP on cell migration at 24 h was examined by wound healing (n = 3). (**B**) The invasive capacity of MH7A cells after treatment with IOP was evaluated by transwell assay (n = 3). (**C**) Flow cytometry assay was performed to assess the apoptotic rate of the MH7A cells. (**D**) Western blot assay was conducted to evaluate the protein expression levels of Bcl-2 and Bax in MH7A cells. Data are represented as mean ± SD. * *p* < 0.05, ** *p* < 0.01 vs. control group; ^#^ *p* < 0.05, ^##^ *p* < 0.01 vs. TNF-α group.

We next investigated the effects of IOP on migration, invasion, and apoptosis of MH7A cells. As shown in Figure 6A,B, stimulation with TNF-α (10 ng/mL) significantly enhanced the migratory and invasive abilities of MH7A cells. Interestingly, treatment with IOP could markedly reduce both the migratory and the invasive abilities of MH7A cells compared with the TNF-α group.

Given that the death of cells could also reduce cell proliferation [27], we assessed apoptosis after drug treatment in MH7 cells. Flow cytometry analysis showed that IOP increased apoptosis of MH7A cells in a dose-dependent manner compared with the TNF-α group (Figure 6C). This was further confirmed by Western blot analysis, where IOP treatment upregulated Bax expression and downregulated Bcl-2 expression (Figure 6D).

### 2.7. IOP Inhibits NF-κB and NLRP3 Inflammasome Signaling Pathways in MH7A Cells

**Figure 7:**
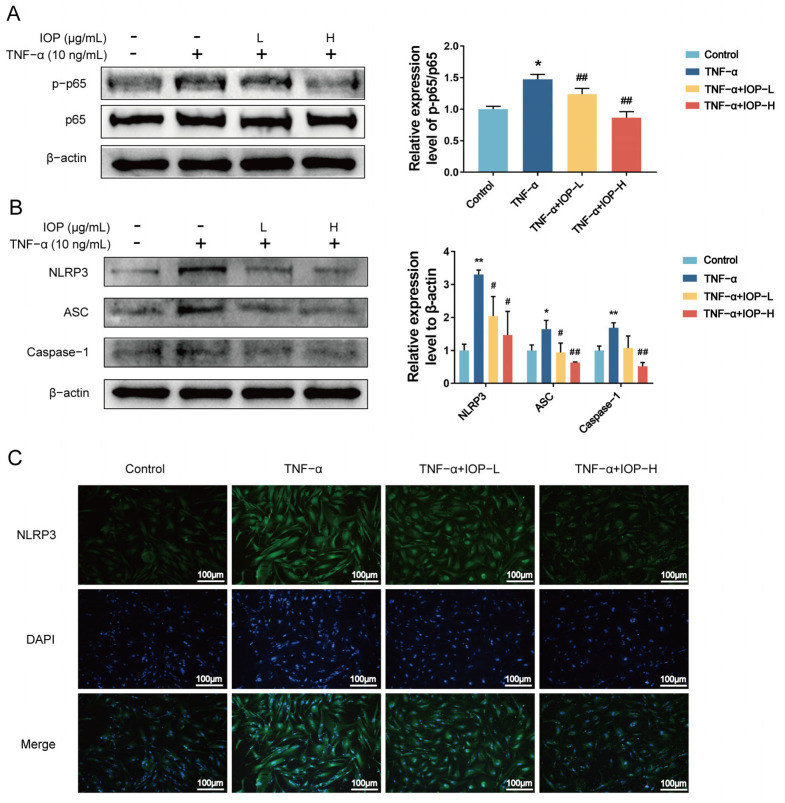

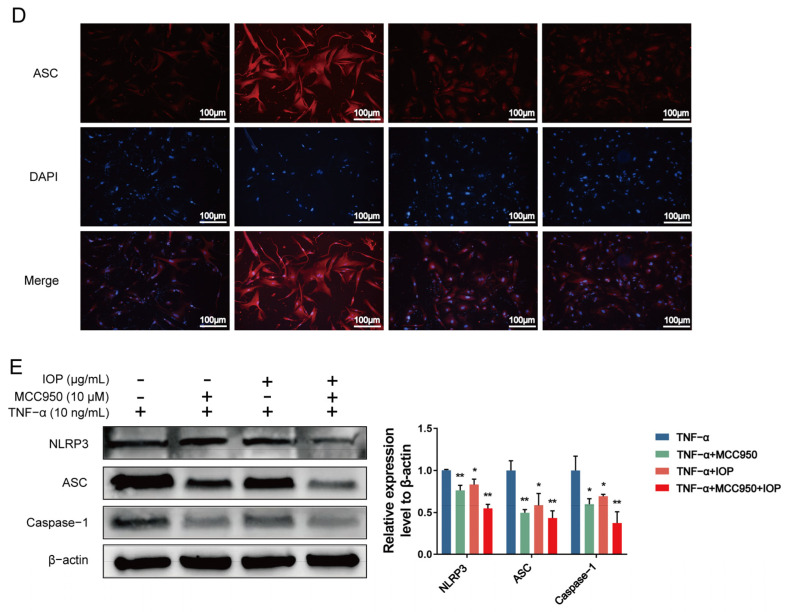
IOP inhibits NF-κB nuclear translocation and NLRP3 inflammasome activation in TNF-α-stimulated MH7A cells. MH7A cells were incubated with IOP (200 and 400 µg/mL) and pretreated with 10 ng/mL TNF-α. (**A**,**B**) Western blot assay was performed to assess the protein expression levels of NF-κB p-p65, NF-κB p65, NLRP3, ASC, and Caspase-1 in MH7A cells. (**C**,**D**) Immunofluorescent staining was conducted to determine the expression of NLRP3 and ASC in MH7A cells. (**E**) Western blot assay was performed to detect the protein expression level of NLRP3, ASC, and Caspase-1 after treatment with NLRP3 inhibitor MCC950 and IOP. Data are represented as mean ± SD. * *p* < 0.05, ** *p* < 0.01 vs. control group; ^#^ *p* < 0.05, ^##^ *p* < 0.01 vs. TNF-α group.

As mentioned before, NF-κB and NLRP3 inflammasome signaling pathways play crucial roles in RA pathogenesis. To further understand the upstream regulatory mechanisms by which IOP modulates MH7A cells proliferation, migration, invasion, apoptosis, and inflammation, we investigated the NF-κB and NLRP3 inflammatory signaling pathways by Western blot and immunofluorescence assays. It is well known that the NF-κB p65 can be activated by phosphorylated p65. As shown in Figure 7A,B, TNF-α promoted the activation of NF-κB p-p65, NLRP3, ASC, and Caspase-1 compared to the control group. Interestingly, IOP treatment suppressed the activation of these proteins. Immunofluorescence results (Figure 7C,D) similarly showed a decrease in the expression of NLRP3 and ASC with increasing IOP concentration. To determine whether IOP exerted an anti-inflammatory effect by inhibiting NLRP3 inflammatory vesicle activation, we used the NLRP3 inhibitor MCC950. The combination of IOP and MCC950 led to a further reduction in the expression of NLRP3, ASC, and Caspase-1. Thus, these data indicate that IOP effectively inhibits the activation of NF-κB and NLRP3 inflammasome signaling pathways, thereby exerting its anti-RA effects.

## 3. Discussion

RA is a chronic autoimmune disease that causes recurrent damage to tiny joints, which may be caused by genetic or environmental factors [28]. Medications for RA include GC, DMARDs like MTX, biological DMARDs, and targeted synthetic DMARDs [29]. However, these drugs often come with significant side effects, including hepatotoxicity, gastrointestinal side effects, carcinogenicity, pulmonary toxicity, hematological toxicity, renal toxicity, and infection. IOP is characterized by low toxicity, low drug resistance, and low side effects as well as a strong anti-inflammatory effect [30,31,32,33]. As mentioned before, previous studies from our lab demonstrated that IOP alleviated inflammatory symptoms in a DSS-induced colitis model by modulating the JAK-STAT pathway [21]. This regulation involved reducing pro-inflammatory cytokines and promoting the balance between Th1/Th2 and Th17/Treg cell populations, contributing to a more controlled immune response. Moreover, our unpublished findings indicate that IOP influences the differentiation of CD4^+^ T cells, reducing the pro-inflammatory Th1 cells and promoting the anti-inflammatory Treg cells, ultimately improving colitis symptoms. Additionally, our previous research confirmed that IOP modulated the NLRP3 inflammasome pathway, inhibiting colorectal cancer progression [16]. Considering the commonality of inflammatory diseases and the results of our previous studies, we were prompted to investigate whether IOP could have a similar therapeutic effect on RA. Therefore, network pharmacology was used to search for potential molecular targets and possible mechanisms between IOP and RA, and the CIA rat model (in vivo) and MH7A cell model (in vitro) were established to explore the effects of IOP on RA and its possible mechanisms.

The C-type lectin receptor and the NOD-like receptor signaling pathways were identified by network pharmacology as the two most enriched pathways. The C-type lectin receptor signaling pathway is a class of receptors that recognize carbohydrates on the surface of pathogenic microorganisms with the participation of Ca^+^, while RA is aseptic inflammation [34]. Therefore, combined with our previous work, we hypothesized that IOP could exert its anti-RA effects by targeting the NLRP3 inflammasome signaling pathway.

Following this network pharmacological research, we conducted further experiments to verify anti-RA effects of IOP in CIA rats. Similar studies have explored other compounds with anti-inflammatory properties. For example, Jolkinolide B was found to inhibit inflammation and bone destruction in CIA rats through the JAK2/STAT3 signaling pathway, while punicalagin demonstrated similar effects by alleviating joint inflammation, cartilage damage, and systemic bone destruction in CIA mice [35,36]. In the CIA model, we found that IOP increased body weight, decreased DAI score, and improved ankle joint thickness in rats compared with the MOD group. This was further confirmed by the pathologic results of H&E staining and safranin O-fast green staining of rat joints, showing reduced inflammation and tissue damage. These clinical and pathological findings suggest that IOP effectively attenuates joint inflammation in CIA rats, supporting its potential as an anti-RA therapy.

RA FLS (MH7A), which is activated by TNF-α stimulation, is a widely used in vitro model of rheumatoid arthritis (RA) for investigating the specific mechanisms of potential therapeutic agents for the condition [37]. TNF-α not only drives RA-FLS proliferation but also enhances their migratory and invasive phenotype by activating NF-κB and MAPK pathways that regulate actin dynamics and cell–matrix adhesion [38]. The in vitro results showed that IOP blocked TNF-α-induced MH7A proliferation, migration and invasion, indicating interference with these motility pathways. Moreover, previous research has shown that the proliferation of RA-FLS and increased secretion of inflammatory cytokines (such as COX-2, iNOS, IL-6, IL-8, IL-1β, and TNF-α) directly contribute to joint destruction [39,40]. In healthy individuals, RA-FLS maintain synovial integrity and secrete fluid to lubricate cartilage surfaces [41]. However, in RA, these cells enhance inflammatory factor secretion and adopt a tumor-like phenotype that promotes migration and invasion, leading to cartilage degeneration and bone damage [42]. The migration of RA-FLS to cartilage and their invasion of the extracellular matrix are fundamental events in RA progression [25]. Additionally, RA-FLS exhibit strong anti-apoptotic properties that accelerate pannus formation [43]. In our study, flow cytometry and Western blot analyses showed that IOP induced apoptosis in TNF-α-stimulated MH7A cells by downregulating Bcl-2 and upregulating Bax. Therefore, targeting the migration, proliferation, and anti-apoptotic functions of RA-FLS is essential for RA therapy, and our findings suggest that IOP could effectively inhibit the aggressive behavior of RA-FLS, thereby contributing to the mitigation of disease progression.

Our study demonstrated that IOP effectively inhibits the activation of NF-κB and NLRP3 inflammasome signaling pathways, both of which are key regulators in RA pathogenesis [8,44]. The suppression of these pathways by IOP was consistent with previous studies involving other compounds such as mangiferin and cinnamic acid, which have also been shown to modulate these pathways [45,46].

Finally, to conduct a more in-depth exploration of the mechanism, we hypothesize that IOP regulates calcium ions, which in turn regulates PKC (protein kinase C)/CaMKII (Calcium–calmodulin (CaM)-dependent protein kinase II), inhibits IKK, thereby inhibiting the translocation of p65 to the nucleus, and thereby inhibiting the assembly of the NLRP3 inflammasome, and ultimately suppressing the inflammation of RA. This will be our future research direction.

## 4. Materials and Methods

### 4.1. Reagents

Main reagents were obtained from the following sources: Recombinant human TNF-α (Sigma-Aldrich, St. Louis, MO, USA); Dulbecco’s Modified Eagle’s Medium (DMEM), fetal bovine serum (FBS), penicillin, and streptomycin (Biological Industries, Beit HaEmek, Israel); CCK-8 kit (Beyotime, Shanghai, China); ELISA kits for human IL-18 and IL-1β (Mei Biao Biological, Shanghai, China); MCC950 (MedChemExpress, Monmouth Junction, NJ, USA); ECL detection reagent (Everbright, San Ramon, CA, USA); anti-NLRP3, anti-caspase-1, and anti-IL-18 (all from Abcam, Cambridge, UK); anti-NF-κB p65, anti-p-NF-κB p65, anti-ASC, anti-IL-1β, anti-IL-6, anti-TNF-α, and anti-β-actin (all from Cell Signaling Technology, Boston, MA, USA); HRP-conjugated goat anti-rabbit and anti-mouse IgG (ZSGB-BIO, Beijing, China); FITC goat anti-rabbit IgG (H + L) and Cy3 goat anti-rabbit IgG (H + L) (APExBIO, Houston, TX, USA).

### 4.2. Preparation of IOP

IOP was obtained from the Department of Immunology and Pathogen Biology of Yanbian University. Our previous study [21] identified IOP through chromatographic characterization using high-performance liquid chromatography [18,47]. The hydrophobic properties (Alogp), oral absorption rates (OB), and drug-likeness (DL) parameters of the main components of IOP were queried in the TCMSP database (https://old.tcmsp-e.com/tcmsp.php, accessed 24 July 2024) [48,49]. Detailed values related to these properties were presented in Supplementary Table S1.

### 4.3. Target Prediction and Network Pharmacology Analysis

The six major monosaccharides in IOP (arabinose, galactose, glucose, mannose, rhamnose and xylose) were converted to 2-D chemical structures and uploaded to PharmMapper (http://www.lilab-ecust.cn/pharmmapper/, accessed 24 July 2024) [50] and Swiss Target Prediction (http://www.swisstargetprediction.ch/, accessed 24 July 2024) [51]. Targets with a normalized fit score ≥ 0.6 were retained as putative IOP targets. RA-associated genes were collected from GeneCards (https://www.genecards.org/, accessed 24 July 2024, relevance score ≥ 10), the Therapeutic Target Database (http://db.idrblab.net/ttd/, accessed 24 July 2024) and OMIM (https://omim.org/, accessed 24 July 2024). IOP targets and RA targets were intersected with VENNY 2.1 (https://bioinfogp.cnb.csic.es/tools/venny/, accessed 24 July 2024) to obtain candidate IOP–RA genes. The overlapping genes were imported into STRING v11.5 (https://string-db.org/, accessed 24 July 2024) with a minimum interaction confidence score of 0.7 to construct a PPI network. The PPI network was visualized in Cytoscape v3.8.2 (https://cytoscape.org/, accessed 24 July 2024) and analyzed with the CytoHubba plug-in (degree algorithm) to rank nodes and select the top ten hub genes. Functional annotation of the candidate genes was performed in DAVID v6.8 (https://david.ncifcrf.gov/, accessed 24 July 2024) for Gene Ontology (GO) enrichment with a significance cutoff of *p* < 0.05 [52]. GO analysis was performed to assess the involvement of the identified genes in BP, CC, and MF. KEGG pathway enrichment was carried out in KOBAS 3.0 (http://kobas.cbi.pku.edu.cn/, accessed 24 July 2024) using a false discovery rate (FDR) < 0.05 [53]. GO and KEGG results were exported to the Bioinformatics platform (http://www.bioinformatics.com.cn/, accessed 24 July 2024) for annotation and graphical visualization [54].

### 4.4. Cell Culture

MH7A cells, an SV40-transformed human rheumatoid arthritis synovial fibroblast line, were purchased from BeNa Cell Collection (BNCC342313). Cells were authenticated by short tandem repeat profiling and confirmed to be free of mycoplasma contamination using the MycoAlert™ Mycoplasma Detection Kit (Lonza, Basel, Switzerland). They were maintained in DMEM supplemented with 10% FBS and 1% penicillin/streptomycin at 37 °C in a 5% CO_2_ humidified incubator. For the in vitro RA model, MH7A cells at passage 5–10 were stimulated with 10 ng/mL TNF-α for 24 h before further assays [37].

### 4.5. Establishment of CIA Model and Treatment of Drugs

Female Sprague-Dawley rats (200 ± 20 g, aged 6–8 weeks) were provided by Eisi Laboratory Animal Technologies LLC with license number SCXK (Ji), 2018-0007. The rats were acclimated to a controlled environment with a temperature of 22 ± 1 °C and humidity of 50–65% and had free access to food and water. After 7 days of adaptive feeding, the model was made with an emulsion mixture of 0.2 mL collagen type II and Complete Freund’s Adjuvant at a 1:1 ratio (*v*/*v*), injected subcutaneously into the tail root at day 0 and 7 [55]. The rats were randomly divided into the following 4 groups (n = 7): control group (sterile physiological saline solution), model (MOD) group, MTX group (1.5 mg/kg/week, total three times), and IOP group (100 mg/kg/day), which were administered by gavage for 15 days from the day of the onset of arthritis. The rats’ weight and paw swelling were measured every seven days during the process of the experiment. Each paw was graded on a scale of 0–4 based on previous reports [56]. The severity of arthritis was assessed using an arthritis index, and the arthritis score was calculated for CIA rats. The protocol of the present study was approved by the Committee on the Ethics of Animal Experiments of Yanbian University (YD20210041). All animal experimental procedures were performed in accordance with the guidelines of the Ethical Committee for the Experimental Use of Animals at Yanbian University (Yanji, China).

### 4.6. Histological Analysis

The ankle joint tissue samples were paraffin-embedded, sectioned (thickness 5 μm), and stained with hematoxylin–eosin (H&E) staining and safranin O-fast green staining. The images were observed using a microscope and histological analysis was performed. Hyperplasia, inflammation, and bone erosion scores were obtained as described in a previous study [57].

### 4.7. ELISA Assay

In vivo blood samples were collected from CIA rats by cardiac puncture, allowed to clot at room temperature for 30 min, then centrifuged at 1500× *g* for 10 min. The serum supernatants were aliquoted and stored at −80 °C until assay. The ELISA kits used were purchased from Shanghai Enzyme-linked Biotechnology Co., Ltd. (Shanghai, China). The concentrations of IL-1β and IL-18 in the serum of CIA rats were determined according to the manufacturer’s instructions [58].

### 4.8. Cell Counting Kit-8 (CCK-8) Assay

MH7A cells (5 × 10^3^ cells/well) were seeded into 96-well plates and treated with different concentrations of IOP (100, 200, 300, and 400 μg/mL) combined with TNF-α (10 ng/mL) for 24 h. After incubation, 10 μL of CCK-8 reagent was added to each well, followed by incubation for an additional 2 h. The absorbance was measured at 450 nm using a Synergy H1 Microplate Reader (BioTek Instruments, Inc., Winooski, VT, USA).

### 4.9. 5-Ethynyl-2′-Deoxyuridine Incorporation Assay

MH7A cells (5 × 10^3^ cells/well) were seeded in 96-well plates, treated with TNF-α and IOP for 24 h, and then fixed and washed. The EdU medium, Click reaction, and Hoechst 33342 (Beyotime, Shanghai, China) were used for cell staining. Fluorescence microscopy (Olympus, Tokyo, Japan) was used to analyze the percentage of EdU-positive cells compared to Hoechst 33342-positive cells.

### 4.10. Colony Formation Assay

MH7A cells (500 cells/well) were seeded into 6-well plates and treated with TNF-α and IOP for 24 h. The medium was then replaced with fresh medium without TNF-α or IOP, and the cells were cultured for 1 week. The cells were stained with Giemsa solution for 15 min, photographed, and the colonies were counted.

### 4.11. Migration and Invasion Assay

MH7A cells (2 × 10^5^ cells/well) were seeded in 6-well plates. After the cells had attached to the wells, a wound was scratched through the cell layer with a sterile pipette tip, and the non-adherent cells were washed away. The cells were cultured with IOP for 24 h and photographed for 0 h, 12 h, and 24 h. The wound size and migration distance were calculated.

For the invasion assay, the cells (5 × 10^3^ cells/mL) were inoculated into a Boyden chamber and treated with IOP. At the same time, 600 μL of DMEM containing 10% FBS and 10 ng/mL TNF-α was placed in the lower chamber as a chemical attractant. After incubation for 24 h, the cells were fixed, stained, photographed, and counted under a microscope.

### 4.12. Apoptosis Assay

MH7A cells (2 × 10^5^ cells/well) were seeded in 6-well plates and treated with IOP for 24 h. Apoptosis was assessed using the Annexin V-FITC/PI Apoptosis Detection Kit (Cat. No. C1062M; Beyotime Biotechnology, Shanghai, China). Briefly, cells were harvested (including floating cells), washed twice with cold PBS, and resuspended in 195 µL Annexin V binding buffer. Five microliters of Annexin V-FITC and ten microliters of propidium iodide were added to each sample, which was then incubated for 15 min at room temperature in the dark. The samples were analyzed immediately by CytoFLEX flow cytometry (Beckman Coulter, Brea, CA, USA) with excitation at 488 nm, acquiring at least 10,000 events per sample [59].

### 4.13. Western Blot Analysis

Tissues and cells were lysed in RIPA lysis buffer. Take 20 mg of knee joint synovial tissue and cut it into small pieces. Add RIPA lysis buffer, then use a tissue grinder to thoroughly grind the tissue. Next, centrifuge at 12,000 rpm for 30 min at 4 °C. Collect the supernatant, quantify it, and prepare the WB sample. The proteins were separated by 8–15% SDS-PAGE gels (VWR, Beijing, China), then transferred to PVDF membranes as described previously [60]. The imprints were incubated overnight at 4 °C with rabbit primary antibodies against human or mouse NF-κB p65, NF-κB p-p65, NLRP3, ASC, Caspase-1, IL-18, IL-1β, TNF-α, IL-6, Bax, Bcl-2, and β-actin. The blots were washed and incubated with secondary antibodies for 1 h. Finally, the ECL reagent was used for detection, and the blots were developed and imaged.

### 4.14. Immunofluorescence

MH7A cells were incubated on glass coverslips, fixed, permeabilized, and then incubated with rabbit primary antibodies against human or mouse NLRP3 and ASC for 12 h. After washing, the cells were further incubated with fluorescent secondary antibodies for 1 h, followed by DAPI staining to visualize the nucleus. Finally, images were analyzed using a fluorescence microscope (Olympus, Tokyo, Japan).

### 4.15. Statistical Analysis

GraphPad Prism version 7.0 software (GraphPad Software, Inc., San Diego, CA, USA) was used for statistical analysis. All experiments were repeated three times. The data from these experiments were expressed as mean ± standard deviation (SD). Student’s *t*-tests and one-way ANOVA were used. The value of *p* < 0.05 is considered statistically significant.

## 5. Conclusions

**Figure 8:**
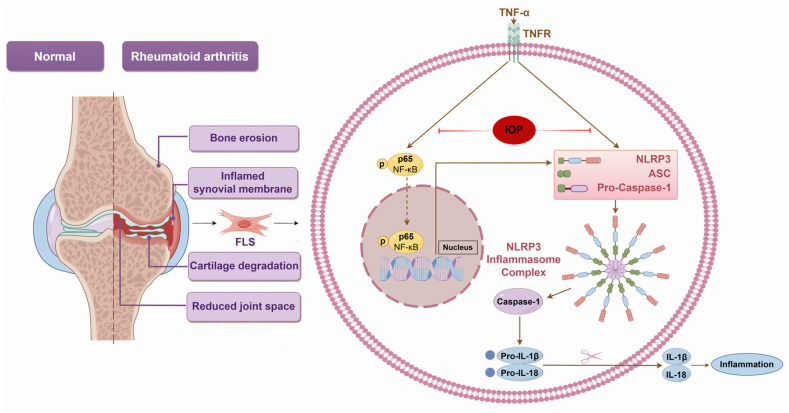
A proposed mechanism underlying of IOP’s therapeutic effects on RA. IOP alleviates arthritis severity and inflammation by inhibiting the activation of the NF-κB and NLRP3 inflammasome signaling pathways. This regulation reduces the production of pro-inflammatory cytokines such as IL-1β and IL-18, thereby mitigating inflammation and joint damage (By Figdraw).

The present study revealed the therapeutic potential of IOP in treating RA, demonstrating its ability to modulate key inflammatory pathways, including the NF-κB and NLRP3 inflammasome pathways, using CIA and MH7A cell models (Figure 8). By inhibiting cell proliferation, migration, and invasion, and promoting apoptosis, IOP could offer a potential therapeutic strategy for alleviating RA.

### Limitations

Although this study provides encouraging evidence for the therapeutic potential of IOP in RA, several limitations must be acknowledged. Firstly, this study only used one type of human rheumatoid arthritis cell (MH7A cells), which may not capture the full heterogeneity of the disease. Future research will consider adding primary cells, such as primary cells derived from RA patients or animal models. Secondly, a detailed structural characterization of IOP, including its glycosidic linkages, branching patterns, and higher-order conformation, remains undefined. To address this limitation, future work will employ one-dimensional and two-dimensional nuclear magnetic resonance spectroscopy, gas chromatography–mass spectrometry of derivatized sugar fragments, and liquid chromatography–tandem mass spectrometry of minor substituents, thereby enabling comprehensive structure–activity relationship studies. Thirdly, while we showed that IOP modulates NF-κB activation and NLRP3 inflammasome assembly, the precise molecular interactions among individual NF-κB subunits and their upstream regulators remain unclear. Therefore, follow-up studies will combine transcriptomics, proteomics, and phosphoproteomics to map these signalling events. Finally, the present work lacks clinical validation. Future investigations will analyze synovial tissues and serum samples from RA patients to correlate IOP exposure with inflammatory markers and clinical outcomes. Despite these limitations, the present findings lay a solid foundation for deeper mechanistic and translational research on IOP as a candidate therapy for RA.

## Figures and Tables

**Table 1:** Targets of IOP against RA.

Number	Gene Symbol	Uniprot ID	Gene Full Name
1	*IL-2*	P60568	Interleukin-2
2	*STAT3*	P40763	Signal transducer and activator of transcription 3
3	*CASP3*	P42574	Caspase-3
4	*TLR4*	O00206	Toll-like receptor 4
5	*KDR*	P35968	Kinase insert domain receptor
6	*MMP2*	P08253	Matrix metalloproteinase-2
7	*ACE*	P12821	Angiotensin-converting enzyme
8	*ABCB1*	P08183	ATP-binding cassette sub-family B member 1
9	*PRKCD*	Q05655	Protein kinase C delta type
10	*CYP2D6*	P10635	Cytochrome P450 2D6

## Data Availability

The original contributions presented in this study are included in the article/Supplementary Materials. Further inquiries can be directed to the corresponding authors.

## Supplementary Materials

The following supporting information can be downloaded at: https://www.mdpi.com/article/10.3390/ph18071017/s1, Figure S1: The six main monosaccharide structural formulas in IOP; Table S1: The Six Monosaccharides in IOP.

## Abbreviations

The following abbreviations are used in this manuscript:

BP	biological processes
CC	cellular components
CIA	collagen-induced arthritis
DAVID	database for annotation, visualization, and integrated discovery
DL	drug-likeness
DMARDs	disease-modifying antirheumatic drugs
DMEM	Dulbecco’s Modified Eagle’s Medium
DSS	dextran sulfate sodium
EdU	5-ethynyl-2′-deoxyuridine
FBS	fetal bovine serum
GC	glucocorticoids
GO	Gene ontology
H&E	hematoxylin–eosin
IL	interleukin
iNOS	inducible nitric oxide synthase
IOP	*Inonotus obliquus* polysaccharide
KEGG	Kyoto Encyclopedia of Genes and Genomes
Man	mannose
MF	molecular functions
MOD	model
MTX	methotrexate
NF-κB	nuclear factor kappa B
NLRP3	NOD-like receptor family pyrin domain containing 3
NOD	nucleotide-binding oligonucleotide domain
OB	oral absorption
PPI	protein–protein interaction
RA	Rheumatoid arthritis
SD	standard deviation
Th1	T helper cell 1
TNF-α	tumor necrosis factor-α
