# Supplementary material for: Integrating Network Pharmacology and Experimental Validation to Explore the Effect and Mechanism of Inonotus obliquus Polysaccharide in the Treatment of Rheumatoid Arthritis
Source: Pharmaceuticals (Basel). 2025 Jul 8;18(7):1017. doi: 10.3390/ph18071017 (PMC12298222; doi:10.3390/ph18071017)
Supplement: Supplementary file 1 [file pharmaceuticals-18-01017-s001.zip › pharmaceuticals-3683703-supplementary.pdf]

Supplementary Material

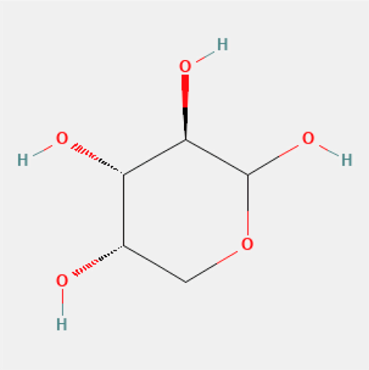

L-Arabinose PubChem CID: 439195
C5H10O5

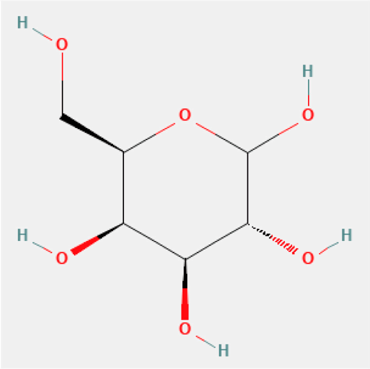

D-Galactose PubChem CID: 6036
C6H12O6

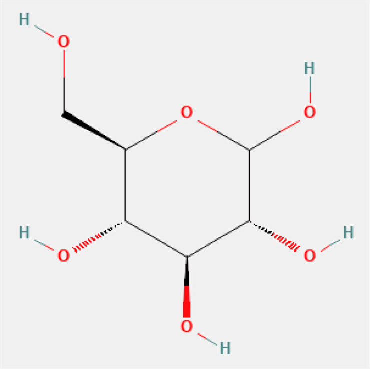

D-Glucose PubChem CID: 5793
C6H12O6

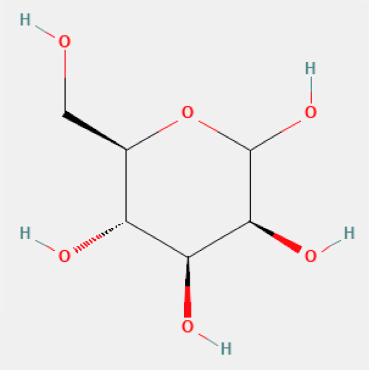

D-Mannose PubChem CID: 18950
C6H12O6

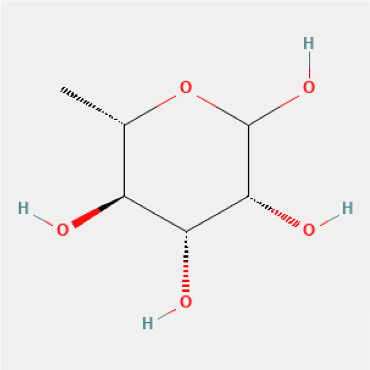

L-Rhamnose PubChem CID: 25310
C6H12O5

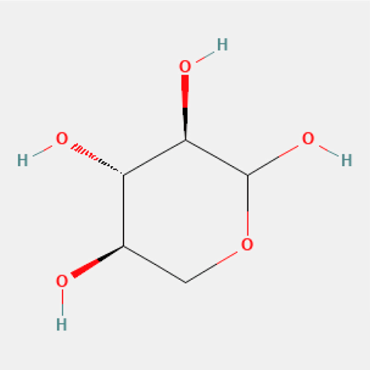

D-Xylose PubChem CID: 135191
C5H10O5

Supplementary Figure S1. The six main monosaccharide structural formulas in IOP.

Supplementary Table S1. The Six Monosaccharides in IOP.

Monosaccharide	Molar Ratio	Alogp	OB(%)	DL
Ara	4.3	-2	54.12	0.03
Gal	11.5	-2.68	47.81	0.03
Glu	46.6	-2.51	50.38	0.04
Man	9.2	-2.51	20.71	0.04
Rha	4.4	-1.62	50.50	0.04
Xyl	11.1	-2.17	51.08	0.02
